# Isometric Resistance Training to Manage Hypertension: Systematic Review and Meta-analysis

**DOI:** 10.1007/s11906-023-01232-w

**Published:** 2023-02-28

**Authors:** B. Baffour-Awuah, M. J. Pearson, G. Dieberg, N. A. Smart

**Affiliations:** Department of Exercise and Sports Science, School of Science and Technology, University of New England, Armidale, NSW 2351 Australia

**Keywords:** Isometric exercise, Isometric resistance training, Blood pressure, Hypertension

## Abstract

**Purpose of Review:**

Hypertension is the primary risk factor for cardiovascular disease and adequate blood pressure control is often elusive. The objective of this work was to conduct a meta-analysis of trial data of isometric resistance training (IRT) studies in people with hypertension, to establish if IRT produced an anti-hypertensive effect. A database search (PubMed, CINAHL, Cochrane Central Register of Controlled Trials, and MEDLINE) identified randomised controlled and crossover trials of IRT versus a sedentary or sham control group in adults with hypertension.

**Recent Findings:**

We included 12 studies (14 intervention groups) in the meta-analyses, with an aggregate of 415 participants. IRT reduced systolic blood pressure (SBP), mean difference (MD) − 7.47 mmHg (95%CI − 10.10, − 4.84), *P* < 0.01; diastolic blood pressure (DBP) MD − 3.17 mmHg (95%CI − 5.29, − 1.04), *P* < 0.01; and mean arterial blood pressure (MAP) MD − 7.19 mmHg (95%CI − 9.06, − 5.32), *P* < 0.0001. Office pulse pressure and resting heart rate was not significantly reduced, neither were 24-h or day-time ambulatory blood pressures (SBP, DBP). Night-time blood pressures, however, were significantly reduced with SBP MD − 4.28 mmHg (95%CI − 7.88, − 0.67), *P* = 0.02, and DBP MD − 2.22 mmHg (95%CI − 3.55, − 0.88), *P* < 0.01.

**Summary:**

IRT does lower SBP, DBP and MAP office and night-time ambulatory SBP and DBP, but not 24-h mean ambulatory blood pressures in people with hypertension.

**Supplementary Information:**

The online version contains supplementary material available at 10.1007/s11906-023-01232-w.

## Introduction

Hypertension is the primary risk factor for cardiovascular disease (CVD) [1]. Adequate blood pressure (BP) control is often not achieved, even with the use of multiple medications and adjunct treatment modalities [2]. The risks of hypertension include physical inactivity, excess dietary salt, being overweight or obese, smoking, and alcohol consumption [3]. Isometric resistance training (IRT) involves sustained contraction against an immovable load or resistance with no change or minimal change in the length of the involved muscle group. It can be performed while seated, without changing clothing, and at any time of day. IRT has been most commonly delivered in the form of unilateral (one arm only) handgrip squeezing activity at 30% of one’s maximum voluntary contraction (MVC) for four bouts of 2 min, with a 3-min rest in-between each squeezing bout, thrice weekly. Historically, people with hypertension avoided IRT due to concerns about hypertensive responses. However, recent work has demonstrated that hypertensive effects during IRT are not as extreme as once thought, and in fact, chronic anti-hypertensive effects have been observed following eight weeks exposure to IRT [4]. IRT may reduce blood pressure by a similar magnitude to taking a single anti-hypertensive medication [5]. The activity does not require much space, requires inexpensive equipment, and elicits less physical stress than aerobic activity. In contrast, the generally recommended exercise prescription for treatment of hypertension would involve 30 min of aerobic exercise at moderate intensity, five times weekly [6].

It is postulated that handgrip exercise either completely or partially occludes the brachial artery, and upon cessation of squeezing, the returning blood flow causes a rebound flow-mediated dilatation of the vessel. Progressive exposure to IRT may therefore enhance the vasodilatory response and in time may even increase the vessel diameter. In normotensive or pre-hypertensive participants the effect of IRT on blood pressure may be smaller due to a reduced potential for non-hypertensives to reduce their blood pressure. Ambulatory blood pressure monitoring (ABPM) over a 24-h period may be more accurate than office-based measurements due to the effects of ‘white coat hypertension’. ABPM is considered the ‘gold standard’ blood pressure measurement technique in some countries [7].

Previous meta-analyses have examined the effectiveness of endurance [8], dynamic resistance training [9], and IRT in lowering resting blood pressure [10] in both healthy and hypertensive populations, but results vary with baseline blood pressure. The findings show isometric resistance exercise does lower blood pressure [11]; however, IRT trials have generally been small, and several are not randomised controlled trials (RCTs) [12••]. Several new IRT trials in people with hypertension have been recently published [13–16]. A recent meta-analysis attempted to quantify IRT elicited blood pressure change from RCTs in people treated for hypertension; however, this pooled analysis incorrectly included one RCT, and some included participants did not meet stipulated inclusion criteria, and other eligible works were overlooked [17•]. We therefore aimed to conduct an updated systematic review and meta-analysis of data from RCTs and randomised crossover trials, that also included recent works published since the previous analysis and evaluated novel outcome data such as day-time and night-time ambulatory blood pressures.

## Methods

This systematic review and meta-analysis followed the Preferred Reporting Items for Systematic Reviews and Meta-Analysis guidelines [18]. The protocol of this work was published by the Cochrane Library: protocol CD 013803 of this work [19].

### Searches

We conducted a systematic search of the literature using both electronic and hand searching methods for randomised controlled and crossover trials of IRT that reported change in blood pressure in adults with hypertension versus a sedentary or sham control group. We searched PubMed, CINAHL, and the Cochrane Central Register of Controlled Trials (CENTRAL) via (CRS-Web) and MEDLINE Ovid (from their inception to July 2, 2022). The subject strategies for databases were modelled on the search strategy designed for MEDLINE (Supplementary Table S1). We contacted experts/organisations in the field to obtain additional information on relevant trials. We contacted original authors for clarification and further data if published information was unclear.

### Study Inclusion and Exclusion Criteria

We included RCTs and randomised cross-over studies comparing IRT with a sedentary or sham control group in adults (≥ 18 years of age), diagnosed with essential hypertension, with resting blood pressure greater than 140/90 mmHg, measured by manual auscultation or automated cuff inflation. We considered studies of IRT at above 10% MVC, versus non-IRT control or sham IRT at an intensity of 10% or less of MVC, delivered for a minimum of 2 weeks or six sessions.

The exclusion criteria were as follows: (1) animal studies; (2) review papers; (3) acute exercise studies; (4) non-RCTs, except for randomised crossover studies; (5) studies that did not report any of the desired outcome measures; or (6) studies that did not have a sedentary or sham control group.

### Outcome Measures

Primary outcomes were change in blood pressure from baseline or after intervention, systolic, diastolic, and mean arterial pressure (measured by manual auscultation), 24-h blood pressure (measured by automated 24-h ambulatory monitoring), and day-time and night-time ambulatory blood pressures. Secondary outcomes were change in pulse pressure and resting heart rate.

### Data Extraction and Synthesis

Two review authors (BBA, MJP) independently assessed all identified articles and consulted a third review author (NAS) to resolve disagreements. Review authors (NAS, MJP) extracted data using an approved data extraction sheet and a review author (GD) resolved any disagreements. We recorded information on outcome measures and archived these in a database. We recorded the following outcome measures: office and ambulatory systolic blood pressure (SBP), diastolic blood pressure (DBP), mean arterial pressure (MAP), and pulse pressure and heart rate (beats/min). If MAP was not reported, it was not calculated because of potential error in calculating pre-post change in standard deviation. In addition to the primary and secondary outcome data, we extracted the following baseline clinical data for IRT and control or sham groups: age, gender, body mass, body mass index, medication use, smoking status, and co-morbid disease. We included data from randomised cross-over trials identified, if the wash-out period was considered long enough to reduce carry-over, no irreversible events such as mortality have occurred, and appropriate statistical approaches were used. We contacted investigators to verify and obtain missing outcome data where possible.

### Measures of Treatment Effect

We undertook meta-analyses only where this is meaningful, i.e., if the treatments, participants, and the underlying clinical question are similar enough for pooling to make sense. Continuous data were analysed and reported using mean difference (MD) with 95%CI, calculated from the change in the mean and SD of outcome measures. We calculated change in pre- versus post-intervention mean by subtracting baseline values from post-intervention values. We calculated change in SD between pre- and post-intervention outcomes using either 95% confidence interval (CI) data for pre-post intervention change for each group or where this was unavailable, actual *P* values for pre-post intervention change for each group. If only the level of statistical significance is available, we used precise *P* values (e.g., P = 0.034), where it was possible for us to obtain these from study authors. Where we were unable to obtain these data, we calculated the change SD from pre- and post- standard deviation data, conservatively assuming a correlation coefficient *R* = 0.5 [20].

We pooled data from each study when four or more studies provided data. We used a random-effects model, even when substantial heterogeneity was absent (*P* value > 0.10, *I*^2^ < 50%) as this provided a more conservative statistical comparison of the difference between intervention and control groups. We completed the data synthesis and analyses using STATA version 16 SE (Statacorp, TX, USA). We intended to conduct the following sub-analyses when the primary analysis was significant (i) arm versus leg training (because arm exercise is likely to elicit greater blood pressure responses due to lower active muscle mass), (ii) unilateral verus bilateral limb IRT, (iii) number of weeks for IRT program duration, and (iv) medicated versus unmedicated participants.

We assessed heterogeneity using the Cochrane Q test, employing a random-effects model. Where eight or more trials were pooled, we created funnel plots, with 5% and 10% significance level contours, using the DerSimonian-Laird model and used the Egger test to explore small study biases for primary outcomes [21].

### Risk of Bias and Study Quality

We assessed study quality and risk of bias using the established TESTEX tool [22]. We also made GRADE-pro summary table of the evidence using the validated tool [23].

## Results

### Search Results

The search produced a total of 6453 results across all databases, with 13 additional records identified by authors. After deduplication 4015 possible studies remained. We excluded 3971 records based upon title and abstract, leaving 44 full text records. We excluded 28 records as they were not randomised controlled trials of IRT versus sedentary control in people with hypertension. A list of excluded studies with reasons is provided (Supplementary Table S2). We excluded 5 records, and all 13 records identified by authors as these were secondary reports. Twelve studies with a total of 415 participants (213 IRT and 202 controls) with hypertension were included in this review [14–16, 24–32] and qualitative analysis (Supplementary Fig. S1).

### Characteristics of Included Studies

Characteristics of included studies are detailed in Table 1. The studies varied in size from 11 to 79 participants. The studies by Farah et al. [26] and Gordon et al. [24] each had two intervention groups listed as Home and Supervised or Laboratory resulting in 14 comparison groups. Two studies had secondary publications of the same participants. Two studies used a randomised crossover design [15, 32], and we used data from both parts of the crossover design for Taylor et al. [32] but only the first part for Nemoto et al. [15] as it was unclear if the wash-out period was sufficient.

**Table 1:** Summary of included study characteristics

**Study**	**Country**	**N IRT (CON)**	**IRT program**	**BP method**
Badrov et al. [27]	Canada	12 (12)	Handgrip, 10 weeks, 3 days/week, 4 × 2 min, 30% MVC with 1 min rest. Bilateral. Venue: 2 sessions in lab, 1 at home	Automated, baseline and 12 weeks
Correia et al. [28]	Brazil	29 (50)	Handgrip, 8 weeks, 3 days/week, 4 × 2 min, 30% MVC with 4 min rest. Unilateral (dominant arm). Venue: lab	Automated, baseline and 8 weeks
Farah et al. [26]	Brazil	18 Home, 14 Lab, 16 Control	Handgrip, 12 weeks, 3 days/week, 4 × 2 min, 30% MVC with 1 min rest. Bilateral. Venue: (1) home (2) supervised lab	Automated, baseline and 12 weeks, 24 h Ambulatory BP
Gordon et al. [24]	USA	8 Lab, 9 Home 5 Control	Handgrip, 12 weeks, 3 days/week, 4 × 2 min, 30% MVC with 1 min rest. Unilateral. Venue: University lab or home	Automated, baseline, 6 and 12 weeks
Gordon et al. [25]	USA	6 (5)	Handgrip, 6 weeks, 3 days/week, 4 × 2 min, 30% MVC with 1 min rest. Unilateral. Venue: University lab	Automated, baseline and 6 weeks
Nemoto et al. [15]	Japan	27 (26)	Handgrip, 8 weeks, at least 3 days/week, 4 × 2 min @close to 30% MVC with 1 min rest. Bilateral. Venue: home	Automated, baseline, 7 and 8 weeks
Okamoto et al. [16]	Japan	11 (11)	Handgrip, 8 weeks, 5 days/week, 4 × 2 min, 30% MVC with 1 min rest. Bilateral. Venue: University exercise lab	Automated, baseline, 4 and 8 weeks
Palmeira et al. [29]	Brazil	15 (16)	Handgrip, 12 weeks, 3 days/week, 4 × 2 min, 30% MVC with 1 min rest. Bilateral. Venue: supervised lab based	Automated, baseline and 12 weeks, 24 h Ambulatory BP
Punia et al. [14]	India	20 (20)	Handgrip, 8 weeks, 3 days/week, 4 × 2 min, 30% MVC with 4 min rest. Bilateral. Venue: Week 1 lab; Weeks 2–8 home	Automated, baseline and 8 weeks
Stiller-Moldovan et al. [30]	Canada	11 (9)	Handgrip, 12 weeks, 3 days/week, 4 × 2 min, 30% MVC with 1 min rest. Venue: 2 weekly sessions in lab, 1 at home	24 h Ambulatory BP, weeks 0, 4 and 8
Taylor et al. [31]	Canada	9 (8)	Handgrip, 10 weeks, 3 days/week, 4 × 2 min, 30% MVC Alternate Hands. Venue: University Wellness Program	Weekly Auscultation
Taylor et al. [32]	UK	24 (24)	Leg Squat, 4 weeks, 3 days/week, 4 × 2 min with 2 min rest @114° ± 19°. Venue: University exercise lab	Automated, baseline and 4 weeks, 24 h Ambulatory BP

*BP* blood pressure, *min* minute/s, *MVC* maximal voluntary handgrip contraction

### Participant Characteristics

Participants varied between studies in terms of body mass index (BMI) classification; two studies had participants of normal BMI; one study had borderline normal/overweight participants; one study had overweight participants; three studies used borderline overweight/obese participants; two studies had obese participants; and three studies provided no BMI data. With respect to participant gender, two studies provided no information [24, 29], while the remaining studies included both males and females. Two studies were of unmedicated participants [16, 32], and the other studies had medicated participants. Smoking information was not available for four studies; the other studies were of non-smokers. Three studies confirmed participants had no co-morbid disease, while this information was unavailable for other studies.

### Exercise Program Characteristics

All but one study used handgrip IRT at 30% of MVC [32]. This study used lower limb wall squats at a joint angle designed to elicit a specific heart rate response, but this method may not precisely reflect a specific MVC%. One study used a method to prescribe MVC that may have been imprecise as it assigned participants to one of four pre-determined workloads, rather than the common practice to periodically assess each person’s MVC and assign them to 30% of maximum load achieved [15]. Study duration varied from 4 to 12 weeks with the most common duration 8 weeks. Session frequency was most often three per week; the only exceptions was five weekly sessions and instruction to participants of ‘at least three per week’ [15].

### Resting Systolic Blood Pressure

**Fig. 1:**
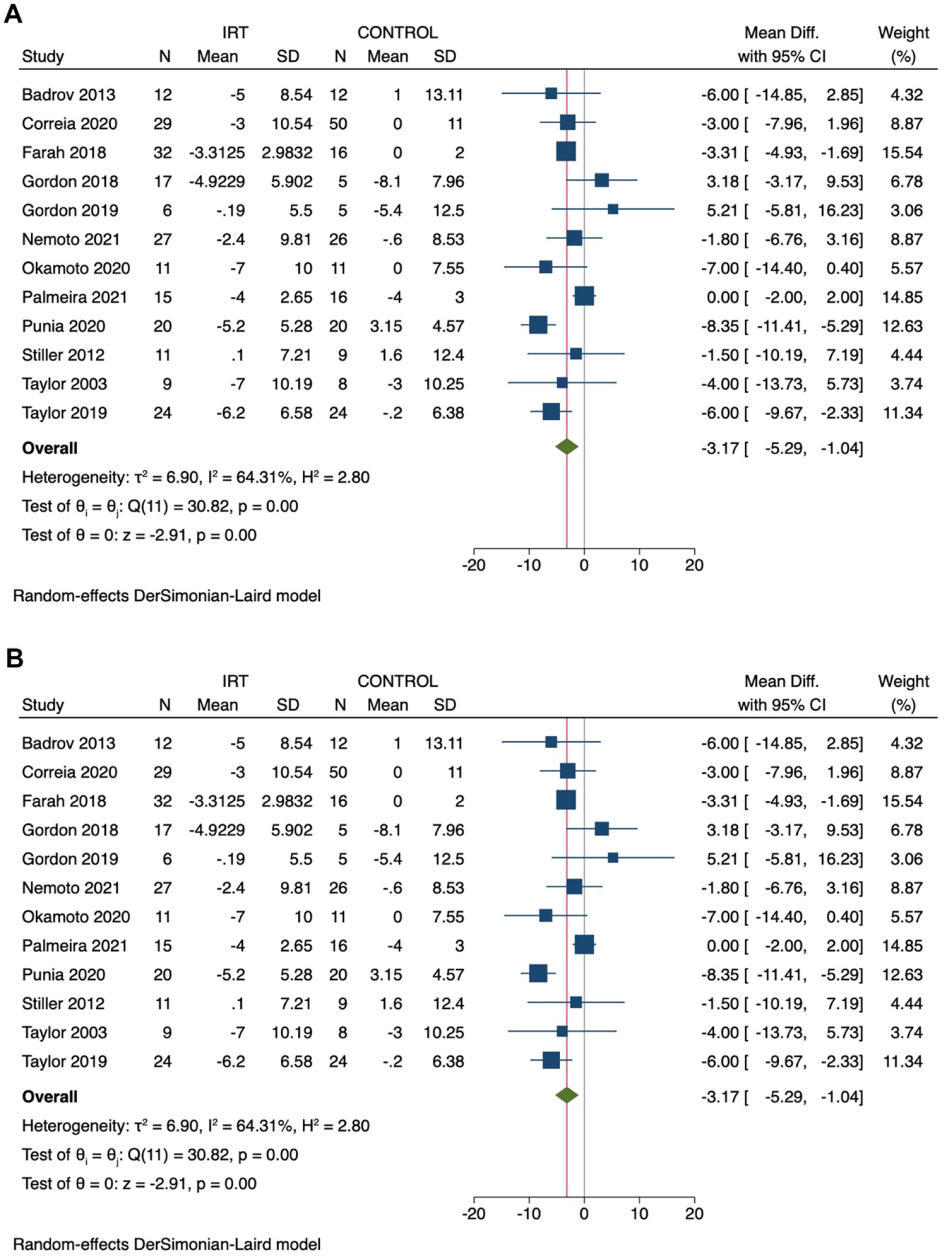
Change in systolic (**A**) and diastolic (**B**) blood pressure: IRT versus control

In a pooled analysis of 12 studies (14 comparison groups – 415 participants), IRT significantly reduced SBP with a mean difference (MD) of − 7.47 mmHg (95%CI − 10.10, − 4.84), *P* ≤ 0.01, *I*^*2*^ = 57% (Fig. 1a).

### Systolic Blood Pressure Sub-analyses

**Fig. 2:**
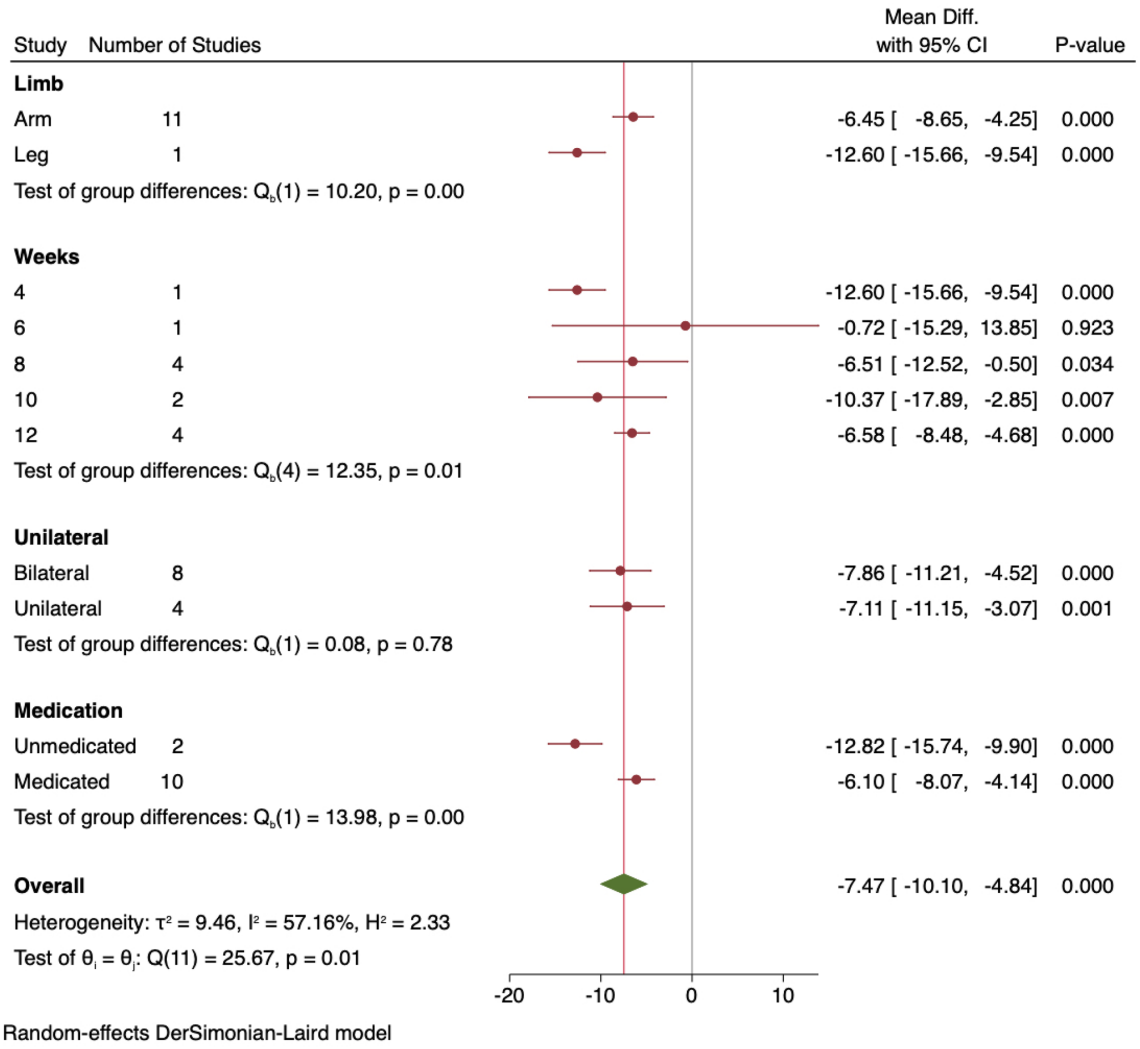
Sub-analysis of systolic blood pressure outcomes: arm vs leg, study duration, unilateral vs bilateral, unmedicated vs medicated

All sub-analyses for SBP can be seen in Fig. 2.

#### Arm Versus Leg IRT

When the one study that used lower limb IRT was removed, the change in SBP for handgrip only IRT was MD − 6.45 mmHg (95%CI − 8.65, − 4.25), and the single study that used leg IRT produced a mean SBP reduction of − 12.6 mmHg (95%CI − 15.66, − 9.54), both *P* < 0.001.

#### IRT Program Duration

IRT for 4, 8, 10, and 12 weeks showed significant reductions in SBP, although the one study of 6 weeks, with only 11 participants, was not significant.

#### Unilateral Versus Bilateral IRT

Both these sub-analyses produced significant and similar reductions in SBP with bilateral IRT participants demonstrating a MD − 7.86 mmHg (95%CI − 11.21, − 4.52), *P* < 0.001, and unilateral IRT participants MD − 7.11 mmHg (95%CI − 11.15, − 3.07), *P* = 0.001.

#### Medicated Versus Unmedicated Participants

Both these sub-analyses produced significant, but similar, reductions in SBP with unmedicated participants showing a MD − 12.82 mmHg (95%CI − 15.74, − 9.90) and medicated participants MD − 6.1 mmHg (95%CI − 8.07, − 4.14), both *P* < 0.001.

### 24-h Mean Ambulatory Systolic Blood Pressure

IRT did not significantly reduce 24-h mean ambulatory SBP; MD − 4.09 mmHg (95%CI − 9.26, 1.09), *P* = 0.12, *I*^*2*^ = 92%, in a pooled analysis of 4 studies (5 comparison groups), with an aggregate of 147 participants (Supplementary Fig. S2).

**Fig. 3:**
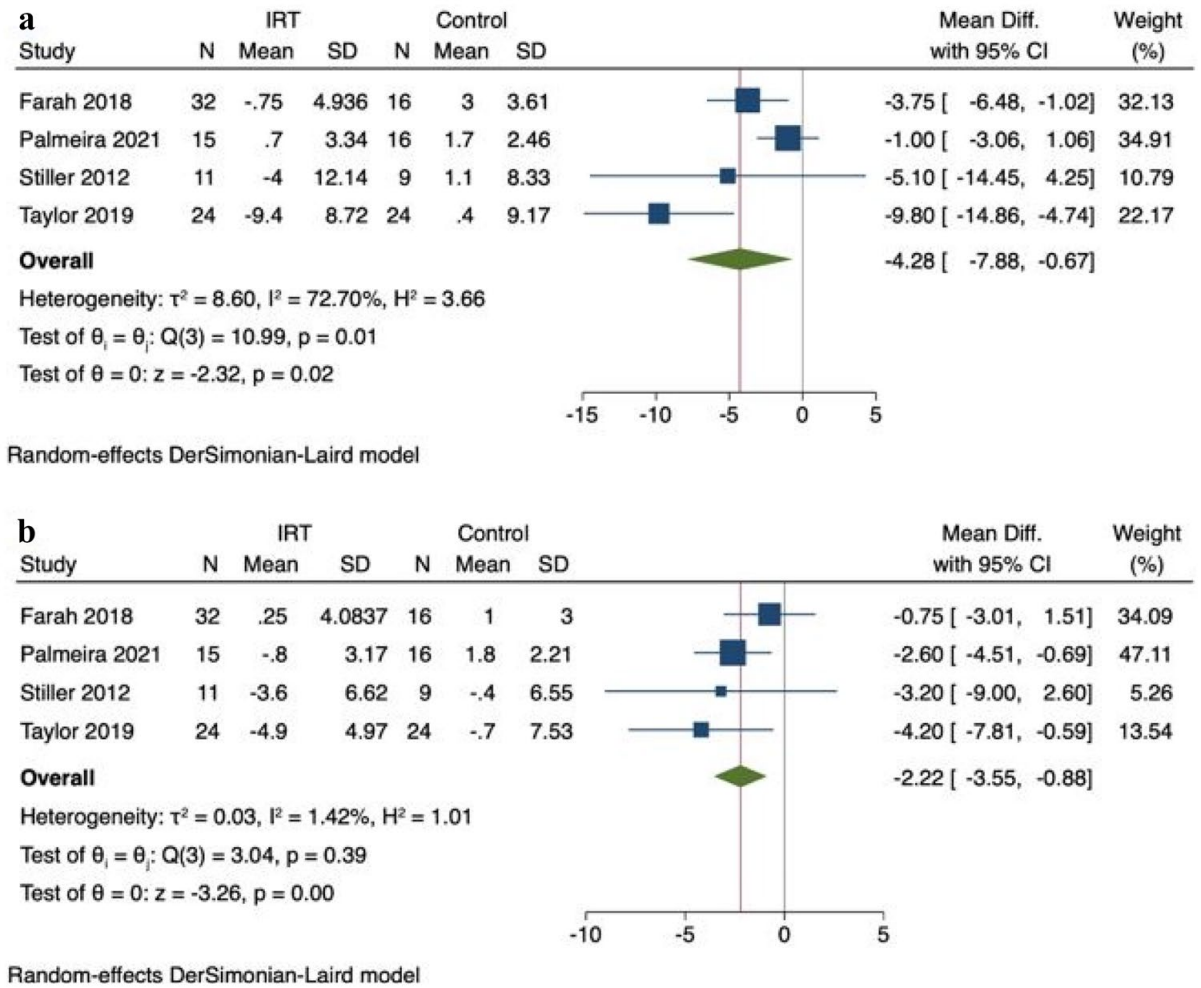
Change in night-time ambulatory systolic (**a**) and diastolic (**b**) blood pressure: – IRT versus control

Day-time ambulatory SBP was not significantly reduced MD − 3.77 mmHg (95%CI − 9.39, 1.84), *P* = 0.19 (Supplementary Fig. S3); however, night-time SBP was significantly reduced MD − 4.28 mmHg (95%CI − 7.88, − 0.67), *P* = 0.02 (Fig. 3a).

### Resting Diastolic Blood Pressure

In a pooled analysis of 12 studies (14 comparisons – 415 participants), IRT significantly reduced DBP with MD − 3.17 mmHg (95%CI − 5.29, − 1.04), *P* < 0.01, *I*^*2*^ = 64% (Fig. 1b).

### Diastolic Blood Pressure Sub-analyses

**Fig. 4:**
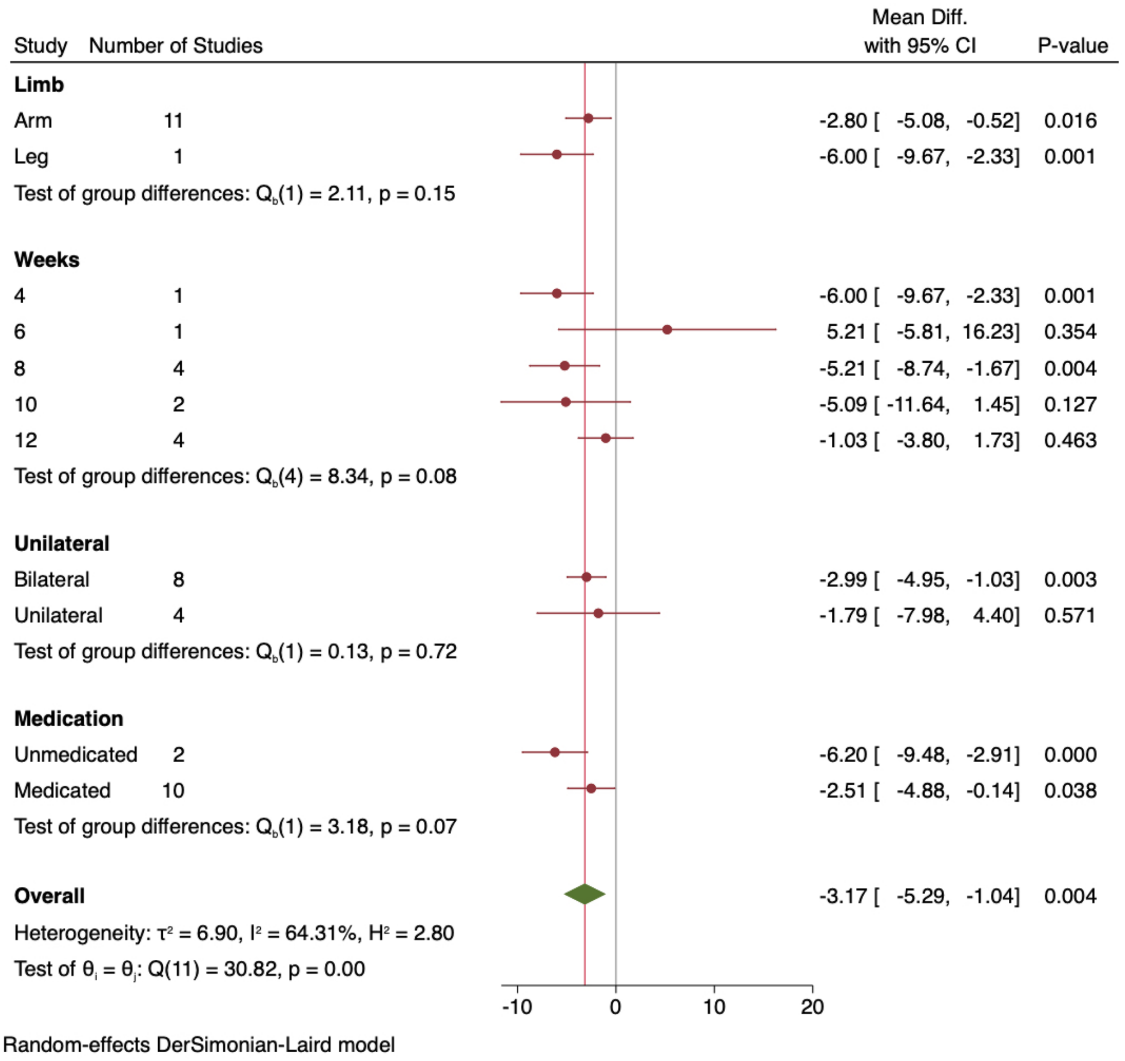
Sub-analysis of diastolic blood pressure outcomes: arm vs leg, study duration, unilateral vs bilateral, unmedicated vs medicated

All sub-analyses for DBP can be seen in Fig. 4.

#### Arm Versus leg IRT

When the one study that used lower limb IRT was removed, the change in DBP for handgrip only IRT was significantly lower with MD − 2.80 mmHg (95%CI − 5.08 to − 0.52), *P* = 0.016, and the single study that used leg IRT produced a DBP reduction of MD − 6.00 mmHg (95%CI − 9.67, − 2.33), *P* = 0.001.

#### IRT Program Duration

IRT for 4 weeks MD − 6.00 mmHg (95%CI − 9.67, − 2.33), *P* = 0.001, and 8 weeks MD − 5.21 mmHg (95%CI − 8.74, − 1.67), *P* = 0.004, showed significant reductions in DBP. Program durations of 6, 10, and 12 weeks were not significant.

#### Unilateral Versus Bilateral IRT

Bilateral IRT participants demonstrated a significant DBP reduction of MD − 2.99 mmHg (95%CI − 4.95, − 1.03), *P* = 0.003, whereas in unilateral IRT participants, the DBP reduction MD − 1.79 mmHg (95%CI − 7.98, 4.40), *P* = 0.57, was not significant.

#### Medicated Versus Unmedicated Participants

Unmedicated participants produced a significant reduction in DBP MD − 6.20 mmHg (95%CI − 9.48, − 2.91), *P* < 0.001, and medicated participants also showed a significant DBP reduction MD − 2.51 mmHg (95%CI − 4.88, − 0.14), *P* = 0.04.

### 24-h Mean Ambulatory Diastolic Blood Pressure

IRT did not significantly reduce 24-h mean ambulatory DBP with MD − 1.98 mmHg (95%CI − 4.53, 0.58), *P* = 0.13, *I*^*2*^ = 70%, in a pooled analysis of 4 studies (5 comparisons), with 147 participants (Supplementary Fig. S4).

Day-time ambulatory DBP was not significantly reduced MD − 1.02 mmHg (95%CI − 2.97, 0.94), *P* = 0.31, *I*^2^ = 48% (Supplementary Fig. S5); however, night-time DBP was significantly reduced with MD − 2.22 mmHg (95%CI − 3.55, − 0.88), *P* < 0.01, *I*^2^ = 1% (Fig. 3b).

### Resting Mean Arterial Blood Pressure

**Fig. 5:**
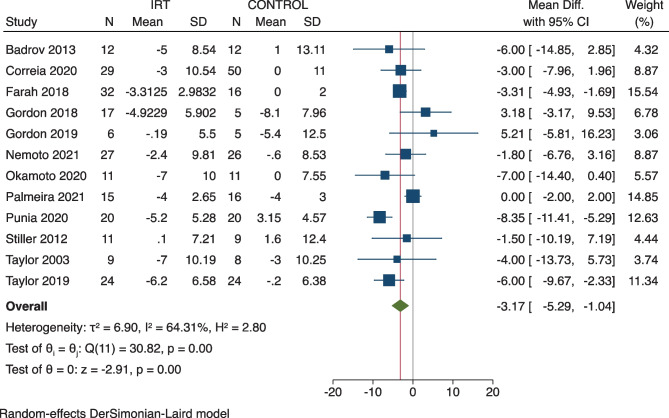
Change in mean arterial blood pressure: IRT versus control

In a pooled analysis of 7 studies (8 comparisons – 184 participants), IRT significantly reduced MAP with MD − 7.19 mmHg (95%CI − 9.06, − 5.32), *P* < 0.0001, *I*^*2*^ = 0% (Fig. 5).

### 24-h Average Ambulatory Mean Arterial Blood Pressure

Only two studies provided this data, and a pooled analysis was not possible.

### Resting Brachial Pulse Pressure

In a pooled analysis of 6 studies (7 comparisons – 261 participants), IRT did not significantly reduce pulse pressure; MD − 1.15 mmHg (95%CI − 2.43, 0.13), *P* = 0.08, *I*^*2*^ = 0% (Supplementary Fig. S6).

### Resting Heart Rate

In a pooled analysis of 8 studies with 294 participants, IRT did not significantly reduce resting heart rate; MD − 1.99 beats/min (95%CI − 4.42, 0.43), *P* = 0.11, *I*^*2*^ = 36% (Supplementary Fig. S7).

### Adverse Events

There were no major adverse events reported.

### Study Quality

Median TESTEX score was 10 (range 9–13); see Table 2. Four items were done poorly in more than 50% of studies: allocation concealment (4 studies), blinding of assessors (3 studies), and activity monitoring in control groups (0 studies) and energy expenditure (0 studies).

**Table 2:** Summary of TESTEX assessment of study quality and reporting of included studies

**Study**	**Eligibility criteria specified**	**Randomisation specified**	**Allocation concealment**	**Groups similar at baseline**	**Blinding of assessor**	**Outcome measures assessed > 85% of participants**^**a**^	**Intention to treat analysis**	**Between group statistical comparisons reported**^**b**^	**Point measures and measures of variability reported**	**Activity monitoring in control group**	**Relative exercise intensity review**	**Exercise volume and energy expenditure**	**Overall TESTEX**
Badrov et al. [27]	1	1	0	1	0	2	1	2	1	0	1	0	10
Corriea et al. [28]	1	1	1	1	1	2	1	2	1	0	1	0	12
Farah et al. [26]	1	1	1	1	0	2	0	2	1	0	1	0	10
Gordon et al. [24]	1	1	0	1	0	2	1	2	1	0	1	0	10
Gordon et al. [25]	1	1	0	1	0	2	1	2	1	0	1	0	10
Nemoto et al. [15]	1	1	1	1	0	2	1	2	1	0	1	0	11
Okamoto [16]	1	1	0	1	1	2	1	2	1	0	1	0	11
Palmiera et al. [29]	1	1	0	1	1	2	0	2	1	0	1	0	10
Punia et al. [14]	1	1	1	1	1	3	1	2	1	0	1	0	13
Stiller-Moldovan et al. [30]	1	1	0	1	0	2	0	2	1	0	1	0	9
Taylor et al. [31]	1	1	0	1	0	2	1	2	1	0	1	0	10
Taylor et al. [24]	1	1	0	1	0	2	1	2	1	0	1	0	10
Total	**12**	**12**	**4**	**12**	**4**	**25**	**9**	**24**	**12**	**0**	**12**	**0**	**Median 10**

Total out of 15 points

^a^Three points possible: 1 point, if adherence > 85%; 1 point, if adverse events reported; 1 point, if exercise attendance is reported

^b^Two points possible: 1 point, if between-group statistical comparisons are reported for the primary outcome measure of interest; 1 point, if between-group statistical comparisons are reported for at least one secondary outcome measure

### Risk of Bias and Heterogeneity

The risk of bias for randomisation was considered low as most groups were matched for key participants characteristics at baseline. As blinding of participants to exercise therapy is not possible, the risk of bias on a possible placebo effect is not possible. As eight of the 12 studies failed to blind investigators to group allocations, there is a possibility of measurement bias.

Heterogeneity was moderate to high for most analyses, but no reasons were identified for this. A possible explanations are study size, manual blood pressure measurement, medication status, and IRT study duration.

### GRADE Recommendations for the Evidence

The GRADE assessment can be seen in Table 3. For two of the five outcomes selected, systolic and diastolic blood pressures, the GRADE recommendation was rated as high certainty of evidence. For MAP as well as night-time ambulatory SBP and DBP, the GRADE recommendation was rated moderate certainty of evidence as the effect size was smaller and rated less important for these outcomes.

**Table 3:** GRADE-pro summary table of IRT compared to control in the management of hypertension

**IRT compared to control in the management of hypertension**					
**Patient or population: **People with hypertension **Intervention: **IRT – Supervised and home-based **Comparison: **Control					
**Outcomes**	Anticipated absolute effects^*^ (95%CI)		**№ of participants** **(studies)**	**Certainty of the evidence** **(GRADE)**	**Comments**
	**Risk with control**	**Risk with IRT**			
Systolic blood pressure	Mean SBP ranged from 8 mmHg lower to 5 mmHg higher	Mean SBP was on average 7.5 mmHg lower (95%CI -10.1 to -4.84 mmHg)	415 (12 RCTs)	⊕ ⊕ ⊕ ⊕ High^a^	SBP is lowered by 7.5 mmHg and cardiovascular event risk is reduced
Diastolic blood pressure	Mean DBP ranged from 8 mmHg lower to 3 mmHg higher	Mean DBP was on average 3.2 mmHg lower (95%CI − 5.29 to − 1.04 mmHg)	415 (12 RCTs)	⊕ ⊕ ⊕ ⊕ High^a^	DBP is lowered by 3.2 mmHg and cardiovascular events risk is reduced
Mean arterial blood pressure	Mean MAP ranged from 4 mmHg lower to 3 mmHg higher	Mean MAP was on average 7.2 mmHg lower (95%CI − 9.06 to − 5.32 mmHg)	184 (7 RCTs)	⊕ ⊕ ⊕ ⊝ Moderate^a^	MAP is lowered by 7.2 mmHg and cardiovascular event risk may be reduced
Night-time systolic blood pressure	Night-time SBP ranged from 0.4 mmHg to 3 mmHg higher	Mean night-time SBP was on average 4.3 mmHg lower (95%CI − 7.88 to − 0.67 mmHg)	147 (4 RCTs)	⊕ ⊕ ⊕ ⊝ Moderate^a^	Night-time ambulatory SBP is lowered by 4.3 mmHg and cardiovascular risk and target organ damage may be reduced
Night-time diastolic blood pressure	Night-time DBP ranged from − 0.7 mmHg lower to 1.8 mmHg higher	Mean night-time DBP was on average 2.2 mmHg lower (95%CI − 3.55 to − 0.88 mmHg)	147 (4 RCTs)	⊕ ⊕ ⊕ ⊝ Moderate^a^	Night-time ambulatory DBP is lowered by 2.2 mmHg and cardiovascular risk and target organ damage may be reduced
*The risk in the intervention group (and its 95% confidence interval) is based on assumed risk in the comparison group and **relative effect** of IRT (and its 95%CI)					
**CI:** confidence interval; **OR:** odds ratio; **RR:** risk ratio					
**GRADE Working Group grades of evidence**					
**High certainty:** We are very confident that the true effect lies close to that of the estimate of the effect					
**Moderate certainty:** We are moderately confident in the effect estimate: the true effect is likely to be close to the estimate of the effect, but there is a possibility that it is substantially different					
**Low certainty:** Our confidence in the effect estimate is limited: the true effect may be substantially different from the estimate of the effect					
**Very low certainty:** We have very little confidence in the effect estimate: the true effect is likely to be substantially different from the estimate of effect					

^a^One study used lower limb IRT. Ages were not given for some studies. Some studies used medicated participants; others were unmedicated

^b^Ambulatory blood pressure, as opposed to office measurements, is considered the gold standard method in some countries

## Discussion

This review is the first meta-analysis to provide a pooled analysis of the effects of IRT on blood pressure, in people with hypertension, in only randomised controlled trials. Our analyses demonstrate that IRT can reduce office, but not mean 24-h or day-time ambulatory, systolic, diastolic, and mean arterial blood pressures in people with hypertension compared to sedentary control. Of note was that night-time systolic and diastolic blood pressures were significanly reduced following IRT.

### Systolic, Diastolic, and Mean Arterial Office Blood Pressures

IRT significantly reduced office systolic, diastolic, and mean arterial blood pressures in people with hypertension by 7.5, 3.2, and 7.2 mmHg, respectively. A program of IRT did not, however, significantly reduce brachial pulse pressure or heart rate, nor 24-h mean ambulatory systolic and diastolic blood pressures. A sub-group analysis of handgrip versus lower limb IRT suggested the latter may produce larger systolic blood pressure reductions, although only 48 participants (1 study) undertook lower limb IRT. These findings are consistent with previous analyses [4, 10, 12••, 33] that have found a 6–10 mmHg reduction in office systolic, 3–6 mmHg reductions in office diastolic, and 1–3 mmHg reduction in mean arterial blood pressures following a program of IRT. The landmark work done by Cornelissen and Smart [33] found a 10.8 mmHg reduction in systolic blood pressure (SBP) and 6–7 mmHg reduction in diastolic blood pressure (DBP); and subsequent works including the robust individual patient data meta-analysis by Smart et al. [12••] showed 7/5 mmHg reductions in SBP/DBP, respectively.

### Effect of Medication on IRT Blood Pressure Response

Medicated participants significantly reduced their SBP by about 6.1 mmHg and those unmedicated by almost 13 mmHg. A similar finding was observed for DBP as medicated and unmedicated participants significantly reduced their DBP by 2.5 and 6.2 mmHg, respectively. One might expect that people with hypertension have greater potential to lower their blood pressure compared to those who are normotensive. In contrast, those with hypertension may be medicated, and therefore the potential to further lower blood pressure may be attenuated by medication. It may be possible that IRT works via a physiological mechanism shared by some antihypertensive medications, but again, this concept must be investigated to further ascertain validity.

### Effect of Exercise Programming Characteristics on IRT Blood Pressure Response

Aside from the single study of 6 weeks IRT, all other program durations yielded a significant reduction in SBP of between 6.5 and 12.6 mmHg. Participants with IRT intervention duration > 8 weeks did not significantly reduce DBP, while those using IRT for 8 weeks or fewer reduced DBP by about 5 mmHg. These finding are counterintuitive and contrast the work of Inder et al. [10] One possible explanation for diminishing blood pressure reductions with longer duration IRT is that participants are more likely to withdraw or become non-adherent to IRT with time.

Participants using bilateral IRT decreased SBP and DBP by 7.9 mmHg and 3 mmHg, respectively, and unilateral IRT participants showed a decrease of 7.1 mmHg and nearly 2 mmHg, respectively, although the small sample of unilateral participants (*n* = 33) is noted. These findings are consistent with the individual patient data meta-analysis by Smart et al. [12••] but contrasted with the findings of Inder et al. [10]

A subgroup analysis of handgrip versus lower limb IRT suggested the latter may produce larger SBP reductions, although only 48 participants from one study undertook lower limb IRT. Handgrip only IRT was significant for DBP change as was lower limb IRT. The small sample of lower limb participants (*n* = 48), from one study, means this finding must be investigated further in a much larger number of participants.

### Systolic and Diastolic Ambulatory Blood Pressures

It is remarkable that neither 24-h ambulatory blood pressures nor day-time ambulatory blood pressures were not significantly altered after IRT; however, night-time ambulatory SBP and DBP were significantly reduced after IRT. Ambulatory blood pressure measures are the gold standard in some regions of the world [7]. One possible explanation for the discrepancy between office and ambulatory outcomes is measurement error, but as all of the included studies used automated devices, this explanation is unlikely. A more plausible explanation is the Hawthorne effect as individuals may alter their behaviour for one-off office measurements but are less able to modify their behaviour over a 24-h period. A second explanation maybe due to the existence of an office versus ambulatory blood pressure difference, which persists during several weeks of antihypertensive treatment, but its magnitude is significantly attenuated [34]. This causes an overestimation of the efficacy of antihypertensive treatment when assessed by only office measures. This overestimation is greater in subjects with an initially greater difference because in these subjects, the subsequent attenuation is greater, or regression to the mean. Because similar phenomena are observed with placebo, the attenuation in the difference during drug treatment is likely to reflect merely habituation to office blood pressure measurements with time [34]. This habituation effect may have been more easily observed in this analysis by those who complete IRT compared to controls as the interaction with researchers is greater.

A previous meta-analysis showed ambulatory blood pressure is more closely related to preclinical target organ damage than office measurements [35]. Also, systolic blood pressure is more closely associated with target organ damage than diastolic, and home blood pressure is as good as ambulatory monitoring and superior to office measurements in regard to association with preclinical organ damage assessed by echocardiography left ventricular mass index [35]. Related to this, other work has suggested the different blood pressure measures assess somewhat distinct parameters; specifically, compared with 3 office visits or 24-h ambulatory, systolic and diastolic home measures over a 1-week period were more reliable and strongly associated with left ventricular mass index [36•].

### Day-Time Versus Night-Time Ambulatory Blood Pressures

It was notable that day-time ambulatory blood pressures were unaltered following IRT, yet night-time systolic and diastolic blood pressures were significantly lower from baseline. Night-time blood pressure and night-time blood pressure decline, termed dipping status, are both considered superior markers of risk of target organ injury and cardiovascular events than values obtained from day-time office blood pressure measurements [37]. A myriad of factors may influence day-time blood pressure measurements, and these are not limited to activities of daily living, fluid and stimulant ingestion, emotional stress, ambient light and noise, circadian variation in neuroendocrine systems, and white coat hypertension [37]. It is perhaps, therefore, unsurprising that continuous monitoring of sleeping blood pressures revealed a benefit that may be at least particlly attributed to IRT.

### Mechanism(s) of Antihypertensive Effect of IRT

Isometric exercise causes an acute stimulation of the metaboreflex in an attempt to restore muscle blood flow [6]. This and other responses to IRT may produce reductions in tissue oxidative stress, improved vascular endothelial function, and favourable changes in baroreflex sensitivity, as well as autonomic balance over the long term [6]. The included studies provided insufficient data to assess oxidative stress and vascular endothelial function. The small number of included studies that provided heart rate variability data were perhaps also insufficient to provide conclusive analyses of baroreflex sensitivity and autonomic balance. The included studies provide some variation in outcome measures; therefore, the precise mechanistic pathways of IRT effect on blood pressure have not been fully elucidated.

### Clinical Implications of Blood Pressure Reductions

The size of the reductions in systolic blood pressure of about 7.5 mmHg and the diastolic blood pressure reduction of 3.2 mmHg are certainly clinically meaningful based upon thresholds reported [12••]. Previous work has suggested IRT elicits a SBP/DBP reduction of (> 5/3 mmHg), which is likely to produce 13–22% reductions in stroke and heart attack, especially in people with uncontrolled hypertension [38]. In terms of healthcare policy, the blood pressure reductions observed with IRT appear to offer the potential to lower mortality, disability, hospitalisation, physician, and medication costs associated with the disease burden from stroke and heart disease. Moreover, the relative cost, accessibility, time requirements, flexibility of venue, and simplicity of the exercise prescription make IRT an attractive and feasible adjunct or alternative to aerobic exercise which has an uptake and adherence to minimum physical activity guidelines in less than 15% of adults in some countries of the developed world [39]. Despite the apparent benefits, IRT continues to be underutilised as an anti-hypertensive therapy; this is possibly because other health benefits such as blood glucose and cholesterol control are not likely with IRT. Another reason that IRT may be underutilised is that blood pressure reductions were significant in office (a one-off measurement in the doctors’ office) and night-time measurements, but not mean 24-h ambulatory or day-time values. Importantly, from a clinical implementation perspective, studies included in the review reported no major adverse events, supporting the safety of IRT as an adjunct therapy in the hypertensive population.

### Limitations and Strengths

The main limitation of this work was between study heterogeneity, which was moderate to high for most analyses, but no obvious reasons were identified for this; however, possible explanations are variations in study size, different blood pressure measurement devices, medication status. and IRT study duration. All included studies provided some outcome data so there was no potential bias from included studies not contributing data. The risk of bias for randomisation was considered low as most groups were matched for participants characteristics at baseline. As blinding of participants to exercise therapy is not possible, an assessment of the risk of bias on a possible placebo effect is not feasible. As four of twelve studies failed to blind investigators to group allocations, there is a possibility of measurement bias, but this was mitigated by using an automated sphygmomanometer to assess blood pressure, which minimises user error or investigator bias.

Nine of the 12 included studies reported 100% adherence to IRT, other studies did not quantify attrition. Low attrition is a strength of this analysis and one of several advantages of IRT over aerobic exercise in terms of adherence and eliciting anti-hypertensive benefits.

### Recommendations for Future Research

Future research should focus on (i) identifying the physiological mechanism of adaptation changes in (a) the renin–angiotensin–aldosterone system or (b) the vascular endothelium; (ii) possible therapeutic uses of IRT in other patient groups beyond hypertension, for example peripheral arterial disease; (iii) identifying, and eliminating from the study design, factors leading to the discrepancy between reductions in resting versus 24-h ambulatory blood pressure measurements; and (iv) investigating the causal link between IRT-elicted reductions in night-time blood pressures and target organ damage and associated cardiac events.

## Conclusion

This review confirms that isometric resistance training produced significant reductions in office systolic, diastolic, and mean arterial blood pressures (7.5, 3.2, and 7.2 mmHg, respectively), but not mean 24-h or day-time ambulatory blood pressures. Night-time ambulatory SBP and DBP were reduced and may lower the risk of target organ injury in people with hypertension.

## Supplementary Information

Below is the link to the electronic supplementary material.

Supplementary file1 (DOCX 1733 KB)
