# Supplementary material for: Isometric Resistance Training to Manage Hypertension: Systematic Review and Meta-analysis
Source: Curr Hypertens Rep. 2023 Feb 28;25(4):35–49. doi: 10.1007/s11906-023-01232-w (PMC10014822; doi:10.1007/s11906-023-01232-w)
Supplement: Supplementary file 1 — Supplementary file1 (DOCX 1733 KB) [file 11906_2023_1232_MOESM1_ESM.docx]

**Supplementary Files**

**SUPPLEMENTARY FILE**


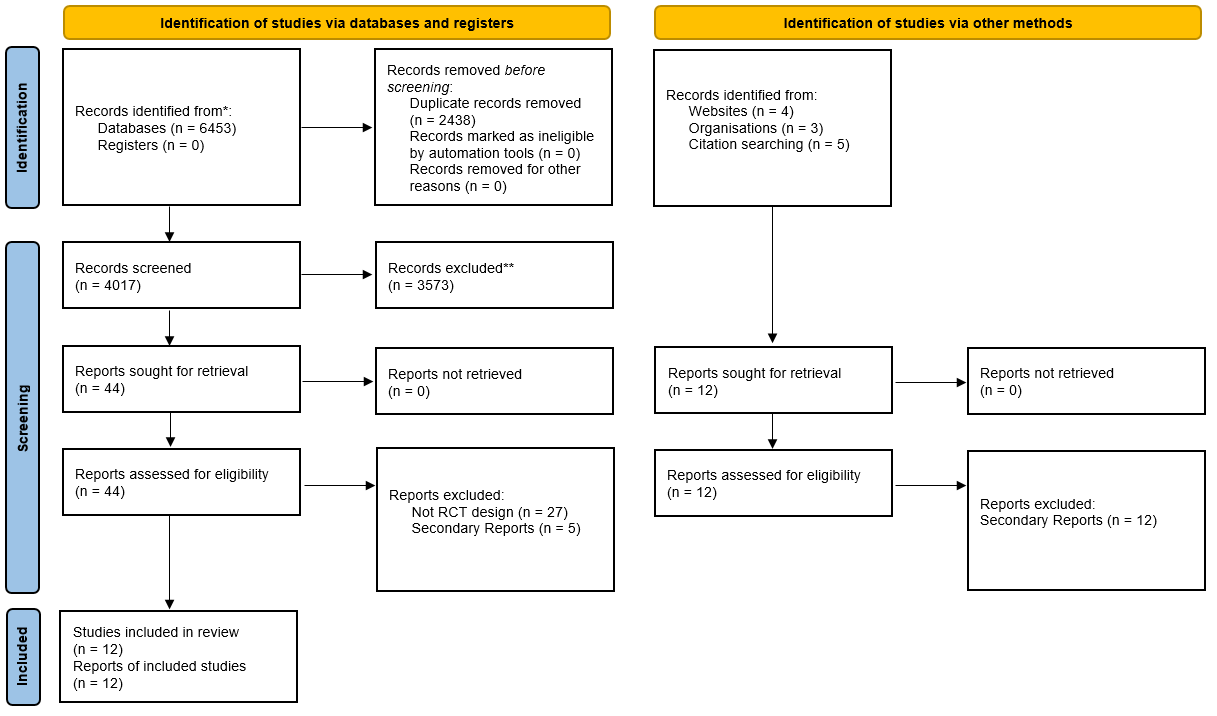


**Figure S1** PRISMA 2020 flow diagram for new systematic reviews which included searches of databases, registers and other sources

**Non-significant pooled analyses – not presented in main text**


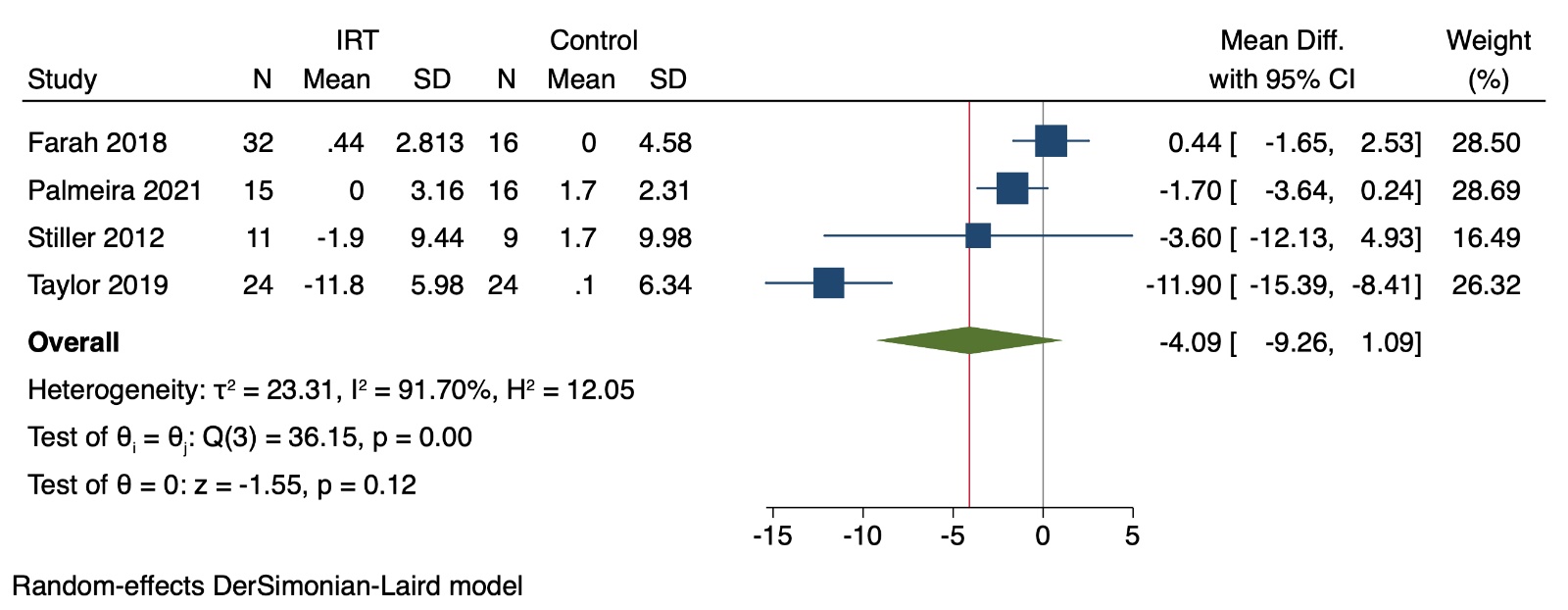


**Figure S2** Change in Ambulatory 24h Systolic Blood Pressure – IRT versus Control


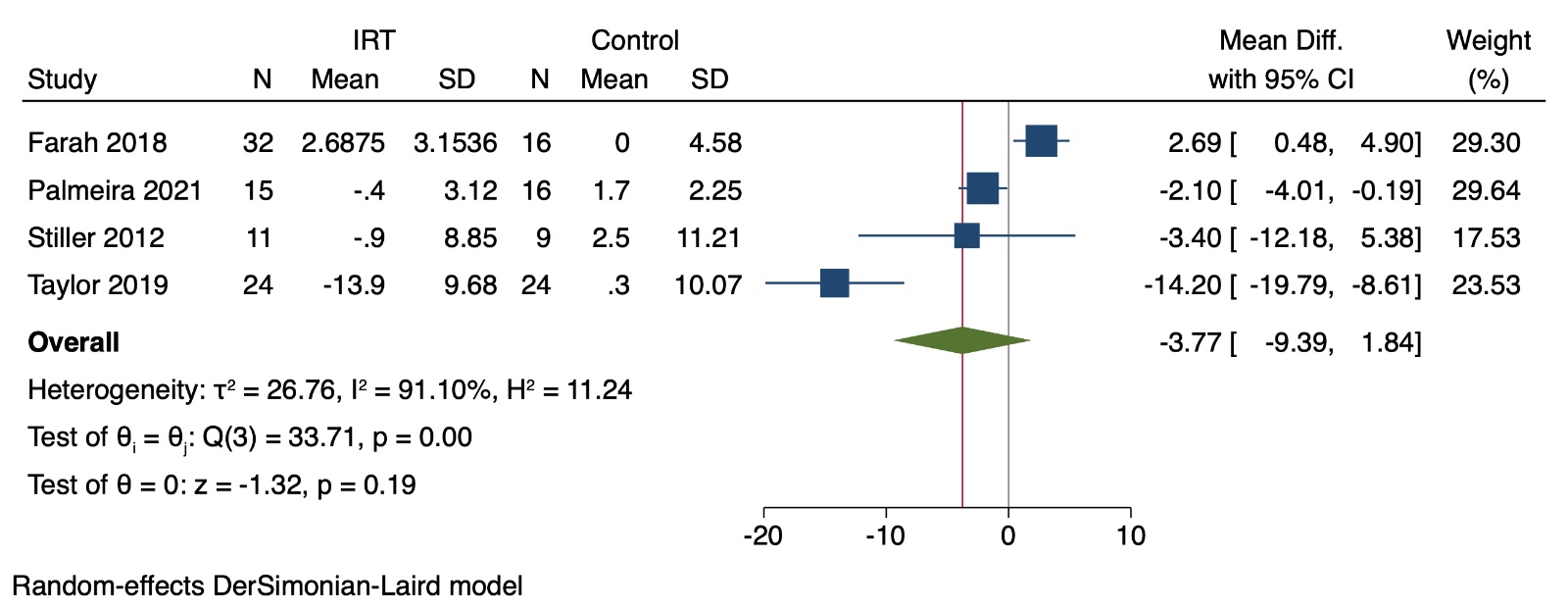


**Figure S3** Change in Day-Time Ambulatory Systolic Blood Pressure – IRT versus Control


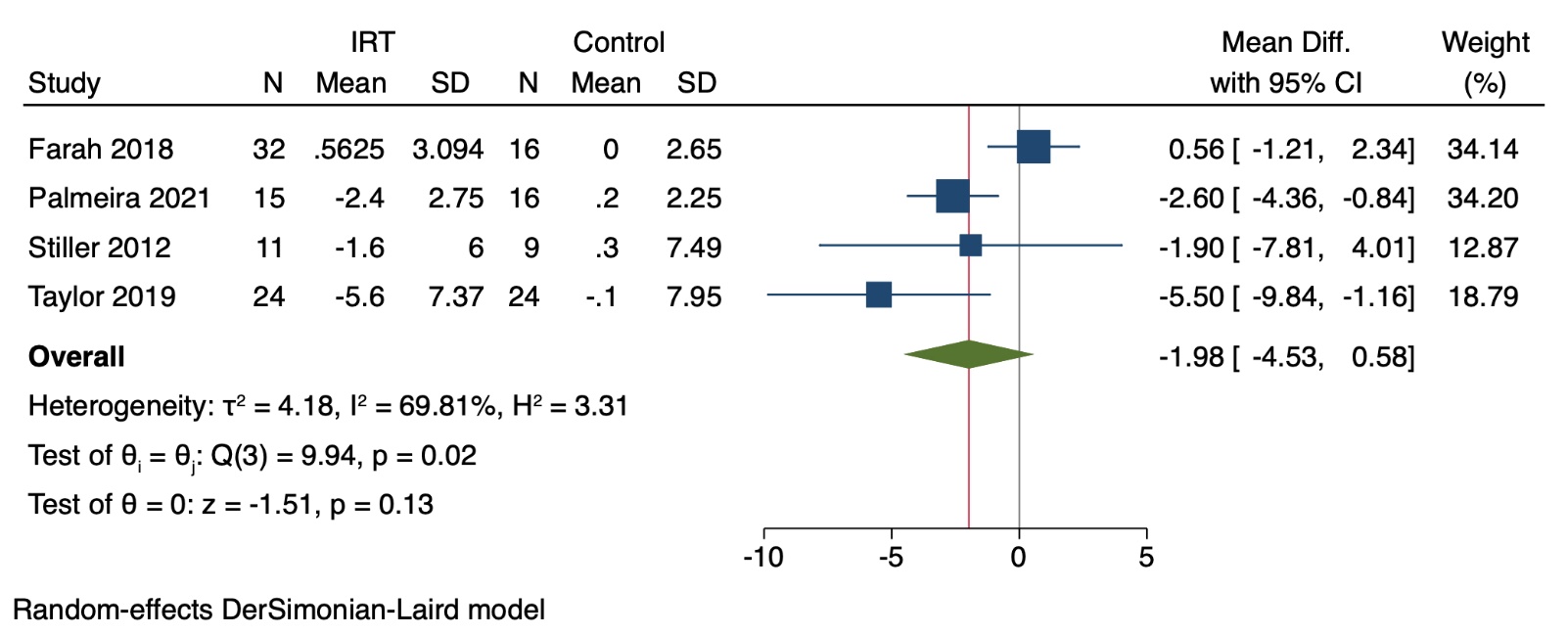


**Figure S4** Change in Ambulatory 24h Diastolic Blood Pressure – IRT versus Control


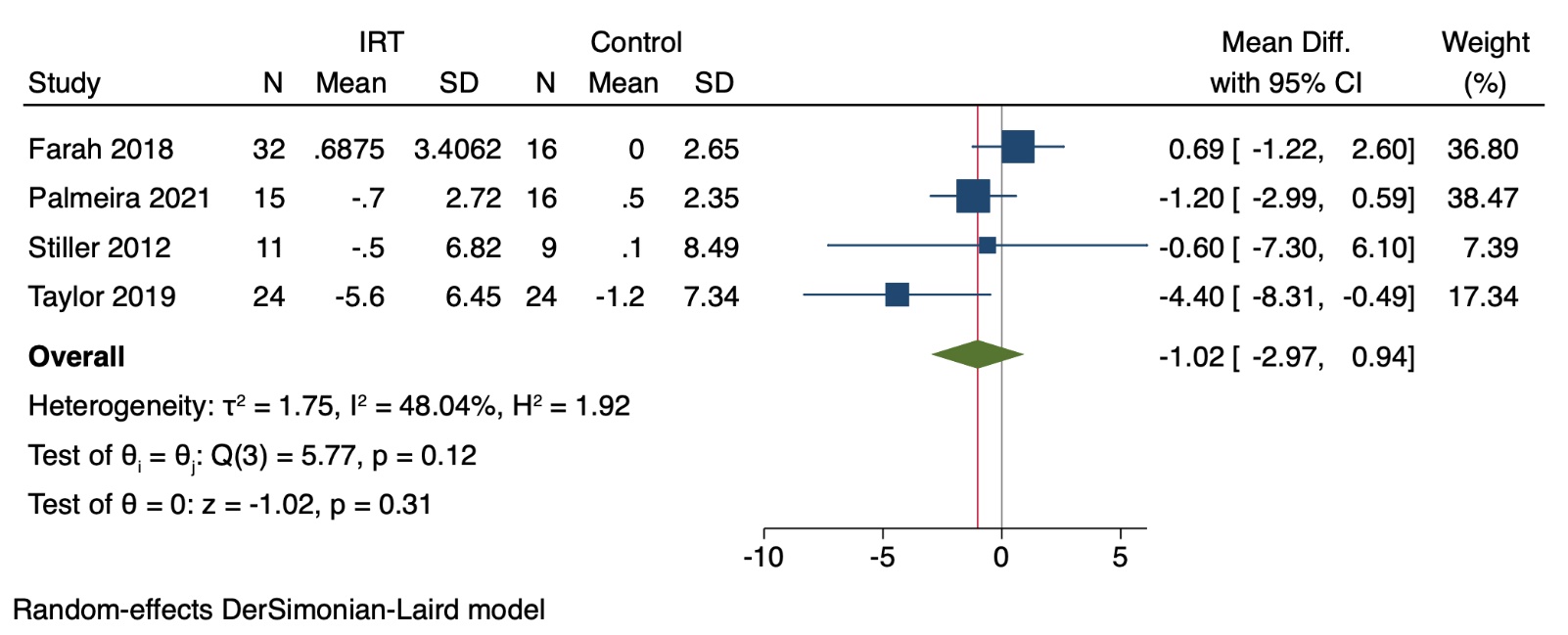


**Figure S5** Change in Day-Time Ambulatory Diastolic Blood Pressure – IRT versus Control


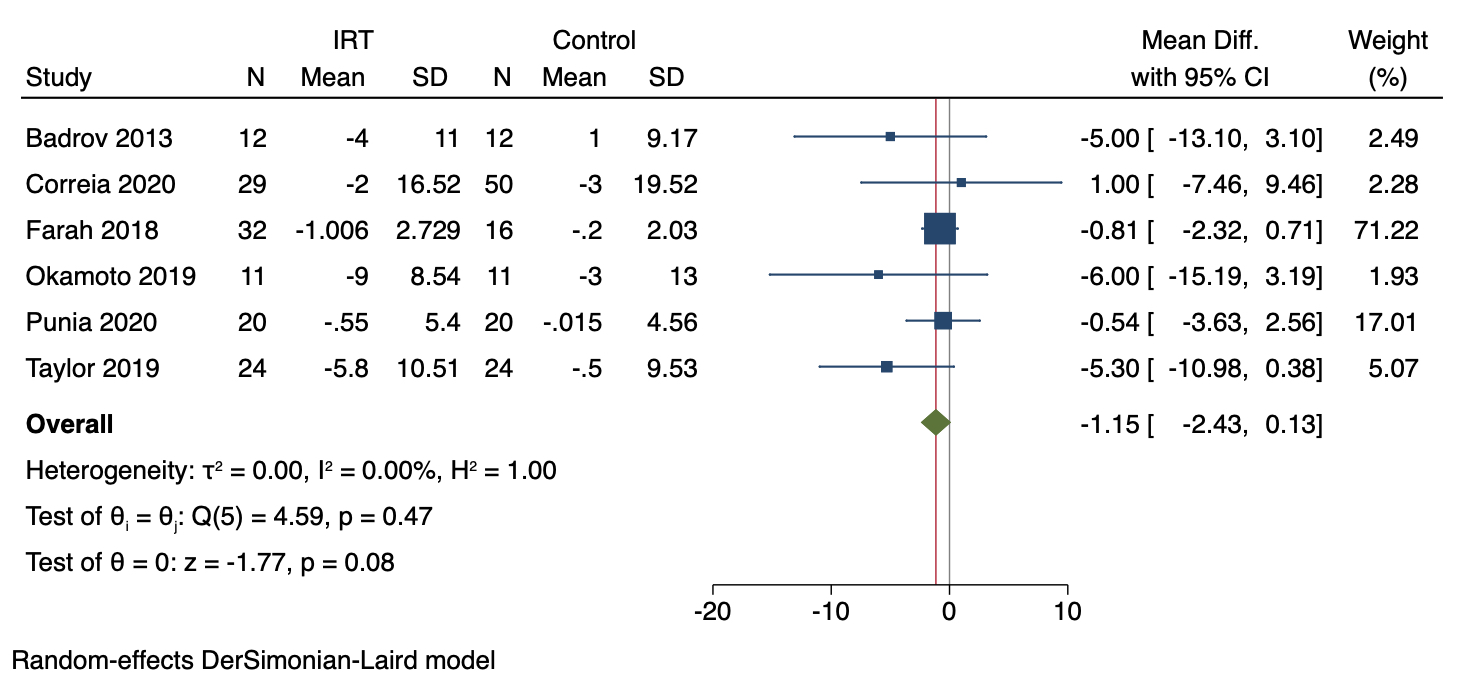


**Figure S6** Change in Pulse Pressure – IRT versus Control


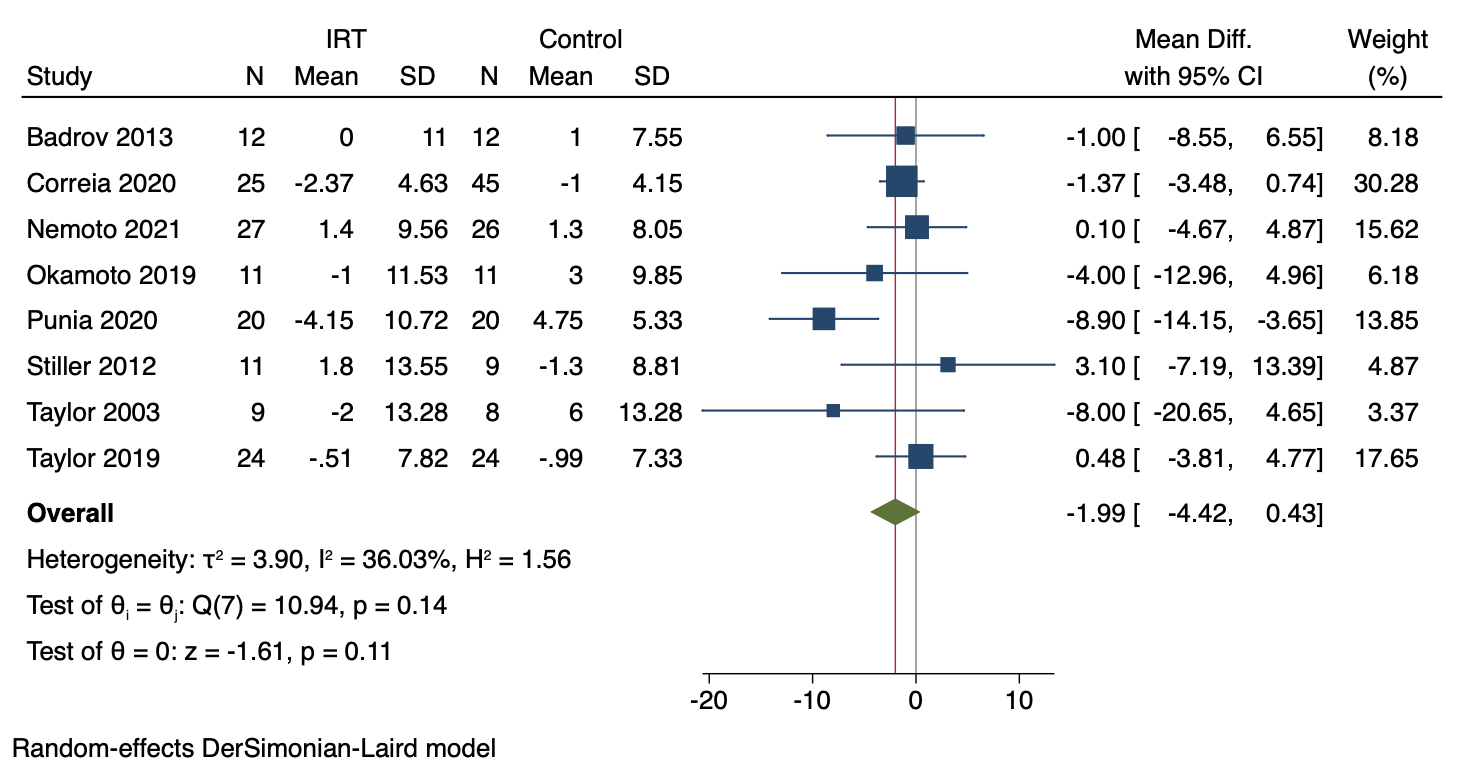


**Figure S7** Change in Heart Rate – IRT versus Control

**Funnel Plots**


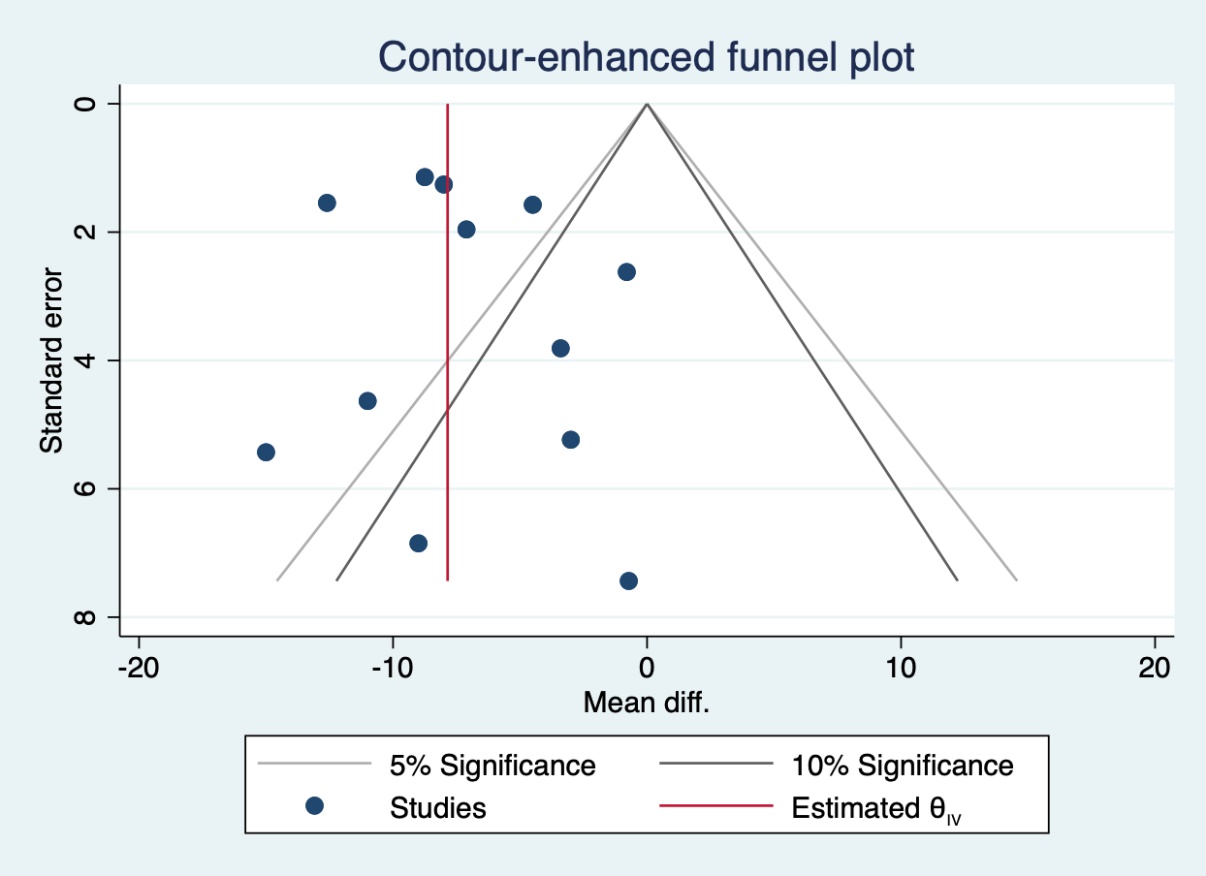


**Figure S8** Funnel plot for Change in SBP – IRT versus Control


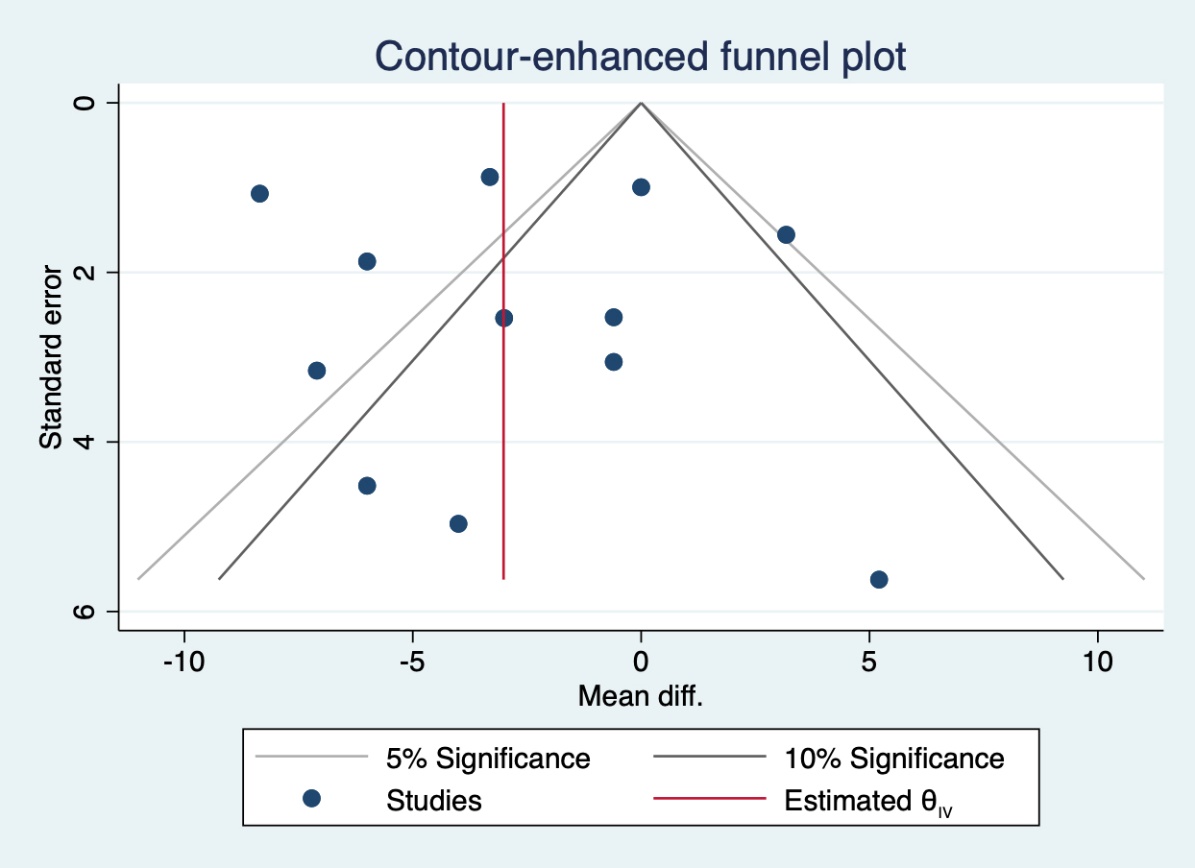


**Figure S9** Funnel Plot for Change in DBP – IRT versus Control


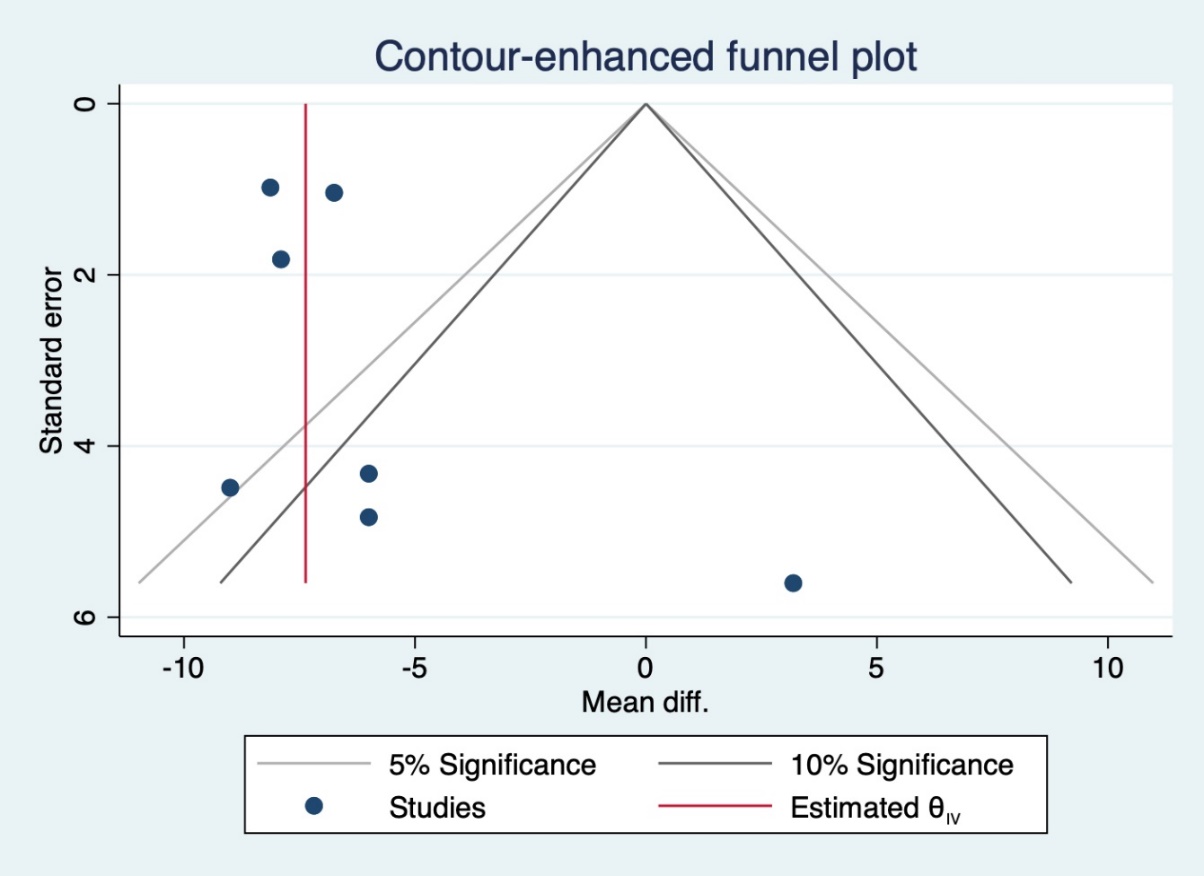


**Figure S10** Funnel Plot for Change in MAP – IRT versus Control


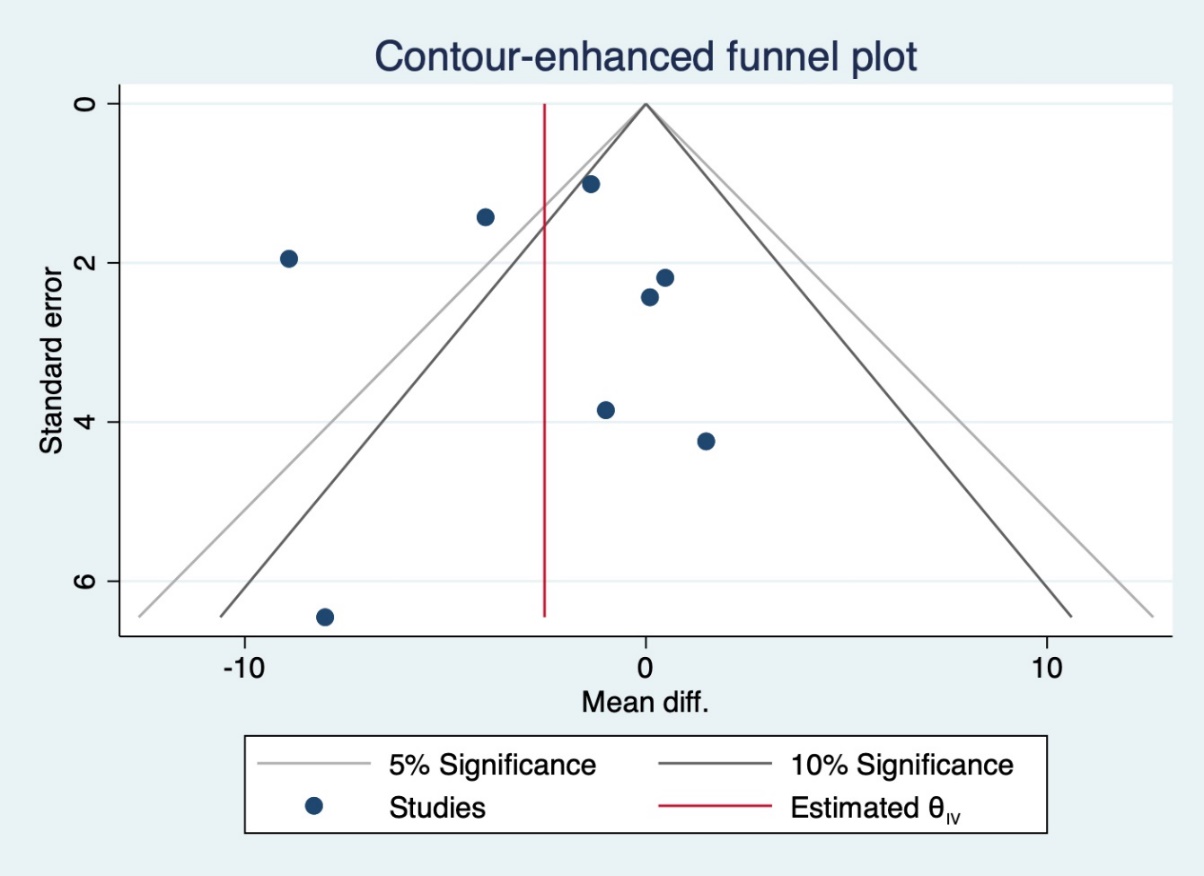


**Figure S11** Funnel Plot for Change in Heart Rate – IRT versus Control

**Table S1** Search Strategy for Ovid MEDLINE(R) ALL 1946 to March 2, 2022

--------------------------------------------------------------------------------
1     exp exercise therapy/ (54747)
2     resistance training/ (9785)
3     isometric contraction/ (15713)
4     ((isometric adj3 contraction*) or (isometric adj3 exercis*) or (isometric adj3 training)).mp. (22668)
5     ((resistance adj3 contraction*) or (resistance adj3 exercis*) or (resistance adj3 training)).mp. (19971)
6     ((strength adj3 contraction*) or (strength adj3 exercis*) or (strength adj3 training)).mp. (10896)
7     ((weight* adj3 contraction*) or (weight* adj3 exercis*) or (weight* adj3 training)).mp. (7932)
8     isometrics.tw,kf. (59)
9     IRT.tw. (3458)
10     or/1-9 (98212)
11     hypertension/ (240848)
12     essential hypertension/ (2416)
13     (antihypertens* or hypertens*).tw,kf. (477540)
14     ((elevat* adj3 blood pressur*) or (high adj3 blood pressur*) or (lower* adj3 blood pressur*) or (reduc* adj3 blood pressur*)).tw,kf. (69557)
15     ((elevat* adj3 bp) or (high adj3 bp) or (lower* adj3 bp) or (reduc* adj3 bp)).tw,kf. (16135)
16     or/11-15 (554035)
17     randomised controlled trial.pt. (532823)
18     pragmatic clinical trial.pt. (1756)
19     controlled clinical trial.pt. (94194)
20     randomi*ed.ab. (624694)
21     placebo.ab. (218585)
22     drug therapy.fs. (2326304)
23     randomly.ab. (358907)
24     trial.ab. (554551)
25     groups.ab. (2203048)
26     or/17-25 (5037396)
27     animals/ not (humans/ and animals/) (4804647)
28     26 not 27 (4382245)
29     10 and 16 and 28 (1575)

**Table S2. Excluded trials of isometric resistance training effects on blood pressure**

| **Study** | **Reason** |
| --- | --- |
| Ash 2017 | Pre-Hypertensive Participants |
| Baddeley-White 2019 | Normotensive Participants |
| Badrov 2013 | Normotensive Participants |
| Baross 2012 | Normotensive/Pre-Hypertensive Participants |
| Baross 2013 | Normotensive/Pre-Hypertensive Participants |
| Barto1 2012 | Duplicate of Stiller-Moldovan 2012 |
| Cahu-Rodriguez 2018 | Duplicate of Farah 2018 |
| Carlson 2016 | Some Pre-Hypertensive Participants |
| Devereux 2011 | Normotensive Participants |
| Gill 2014 | Normotensive Participants |
| Goessler 2016 | Normotensive Participants |
| Goessler 2018 | Normotensive Participants |
| Gordon 2017a | Conference Abstract (full data available in Gordon 2018) |
| Gordon 2017b | Conference Abstract (full data available in Gordon 2019) |
| Hess 2016 | Normotensive Participants |
| Howden 2002 | Normotensive Participants |
| Millar 2008 | Normotensive Hypertensive |
| Ogbutor 2019 | Pre-Hypertensive Participants |
| Oliveira  2021 | Duplicate of Correia 2020 |
| Pagonas 2017 | Aerobic Exercise Intervention also Used |
| Ray 2000 | Normotensive Participants |
| Seidel 2021 | Different Outcome Measures |
| Silva 2018 | Acute IRT (not IRT training program) |
| Silva 2019 | Normotensive Participants |
| Wiles 2010 | Normotensive Participants |
| Wiles 2017 | Normotensive Participants |
| Wiley 1992 | Normotensive Participants |
| Yamagata 2020 | Normotensive Participants |
